# A 10-year trend in the incidence of retinopathy of prematurity in Sichuan, China

**DOI:** 10.3389/fped.2026.1806050

**Published:** 2026-04-08

**Authors:** Wentao Dong, Jiyuan Zhong, Jie Li, Ke Hu

**Affiliations:** 1 Department of Ophthalmology, Sichuan Provincial People's Hospital, School of Medicine, University of Electronic Science and Technology of China, Chengdu, China; 2 Department of Ophthalmology, The First Affiliated Hospital of Chongqing Medical University, Chongqing, China

**Keywords:** incidence, retinopathy of prematurity, screening cost-effectiveness, screening threshold, sepsis, trends

## Abstract

**Objective:**

To analyze the 10-year incidence trend of retinopathy of prematurity (ROP) in Sichuan, China, and explore associated clinical factors and screening strategies.

**Methods:**

In this single-center retrospective study, 603 premature infants undergoing ROP screening between 2014 and 2023 were included. Incidence rates were calculated across five biennial periods, stratified by birth weight and gestational age. Cochran-Armitage test assessed trends; a decision model evaluated screening thresholds; correlation/regression analyses identified clinical associations.

**Results:**

Overall ROP incidence was 19.2% (116/603); Type 1 ROP incidence was 5.1% (31/603). ROP incidence declined from 22.8% to 16.5%, and Type 1 ROP from 5.9% to 4.2% over the study period. No Type 1 ROP occurred in infants >1,500 g or >32 weeks after 2018, with a statistically significant downward trend (*P* < 0.05). Sepsis/bacteremia incidence positively correlated with ROP (r = 0.83, *P* < 0.01), while oxygen therapy compliance (r = −0.89, *P* < 0.01) and breastfeeding (r = −0.76, *P* < 0.05) showed negative correlations. Sepsis/bacteremia was an independent risk factor (*β*=0.45, *P* < 0.01). Adjusting the screening threshold to <1,500 g maintained a Type 1 ROP detection rate of 96.8% while reducing screening costs by 32.5% and false positives by 28.3%.

**Conclusion:**

ROP incidence in Sichuan declined significantly over the past decade. Sepsis is a key risk factor, while improved oxygen therapy and breastfeeding are protective. Adjusting the screening threshold to <1,500 g may enhance screening cost-effectiveness for tertiary medical centers in Sichuan while maintaining a high detection rate for severe ROP.

## 1 Introduction

Retinopathy of prematurity (ROP), a proliferative retinal vascular disorder affecting premature infants and those with low birth weight, is the most common cause of blindness and visual impairment in infants and young children (1, 2). In China, the issuance of the “Guidelines for the Use of Oxygen in the Treatment of Premature Infants and the Prevention and Treatment of Retinopathy of Prematurity” by the National Health Commission in 2004 (3) has actively promoted ROP screening initiatives, effectively reducing the incidence of severe ROP (4). However, disparities in economic development and healthcare service levels across regions have led to inconsistent progress in screening. In economically developed areas such as Beijing, Shanghai, Guangzhou, and Shenzhen, ROP detection rates have exhibited a steady downward trend, attributable to the smooth implementation of screening programs. In contrast, the epidemiological trends of ROP in relatively underdeveloped regions remain inadequately elucidated (5). To fill this knowledge gap regarding regional variations, we conducted a retrospective analysis of ROP screening data from Sichuan Province over the past decade to assess its incidence trends. The results are reported as follows.

## 2 Materials and methods

### 2.1 Study design and participants

This retrospective study enrolled 603 premature infants who underwent ROP screening at Sichuan Provincial People's Hospital between January 2014 and December 2023. The study was conducted in accordance with the tenets of the Declaration of Helsinki and approved by the Medical Ethics Committee of Sichuan Provincial People's Hospital. Written informed consent was waived (No. 2023-563).

### 2.2 Methods

#### 2.2.1 Inclusion and exclusion criteria

Inclusion criteria were based on the “Chinese Guidelines for Screening of Retinopathy of Prematurity (2014)” (1) and included: (1) Premature infants or low birth weight infants with a birth weight < 2,000 g or a gestational age < 32 weeks; (2) Infants with severe systemic diseases or a definite history of prolonged oxygen therapy who were deemed high-risk by pediatricians.

Infants were excluded if they met any of the following criteria: (1) Incomplete clinical data; (2) Failure to complete follow-up until full retinal vascularization or regression of lesions as required by the guidelines due to various reasons; (3) Referral from other hospitals solely for ROP treatment; (4) Presence of other ocular pathologies that might affect vision.

#### 2.2.2 Examination protocol

All participants underwent fundus examination after pupillary dilation using either binocular indirect ophthalmoscopy or a widefield retinal imaging system (RetCam III) in accordance with standard operational procedures. Demographic and clinical data, including birth weight, gestational age, sex, oxygen therapy duration and concentration, feeding method, prenatal glucocorticoid use, and diagnosis of infectious diseases (sepsis, bacteremia confirmed by blood culture or clinical diagnosis), were collected in detail. Fundus examinations were performed by two ophthalmologists with long-term experience in ROP screening and management, in compliance with the “Chinese Guidelines for Screening of Retinopathy of Prematurity (2014)” (1). The diagnosis was recorded based on the most severe condition observed during the entire screening and follow-up period. For asymmetric bilateral involvement, data from the more severely affected eye were used for analysis. Indications for treatment were Type 1 ROP, which included: zone I stage 1+, zone I stage 2+, zone I stage 3, zone I stage 3+, zone II stage 2+, or zone II stage 3 + .

#### 2.2.3 Trend analysis

To evaluate temporal trends in ROP incidence from 2014 to 2023, the study period was divided into five consecutive 2-year intervals: 2014–2015, 2016–2017, 2018–2019, 2020–2021, and 2022–2023. Incidence rates of ROP in each interval were calculated and analyzed stratified by birth weight and gestational age to assess the trend. For the subgroups of birth weight >1,500 g and gestational age >32 weeks, the number of screenings, ROP cases, and Type 1 ROP cases in each time period were counted separately.

#### 2.2.4 Decision analysis model

We established a decision analysis model to simulate the detection rate of Type 1 ROP, screening cost, and false positive rate under three different birth weight screening thresholds: <1,500 g, <1,750 g, and <2,000 g (current threshold). The screening cost includes specific components: fundus examination fee (300 yuan/case, single time), personnel cost (200 yuan/case), and follow-up cost (900 yuan/case, based on the requirement of clinical guidelines that routine screening should be performed at least 3 times on average). The total absolute cost of the current threshold (<2,000 g) is set as 1,400 yuan/case (defined as 100 relative value). The false positive rate refers to the proportion of infants who are screened positive but do not actually have ROP or do not require treatment.

#### 2.2.5 Analysis of influencing factors for incidence

We collected data on changes in clinical practices in each time period, including sepsis/bacteremia incidence (proportion of infants diagnosed with sepsis or bacteremia), oxygen therapy compliance (proportion of infants whose oxygen therapy duration and concentration meet the guidelines), breastfeeding rate (breastfeeding data were extracted from medical records, reflecting the primary feeding method during the first six months postpartum), and prenatal glucocorticoid use rate (proportion of pregnant women who receive prenatal glucocorticoid injections to promote fetal lung maturation). Correlation analysis and regression analysis were used to explore the relationship between these factors and ROP incidence trends.

### 2.3 Statistical analysis

Descriptive statistics were used to analyze the trend of ROP incidence. Further statistical analyses were performed using SPSS 26.0. The normality of continuous variables was tested using the Shapiro–Wilk test. Normally distributed data are presented as mean ± standard deviation (mean ± SD), while non-normally distributed data are summarized as medians. Cochran-Armitage test was used to verify the statistical significance of the trend of Type 1 ROP incidence in specific subgroups. Pearson correlation analysis was used to explore the correlation between clinical practice indicators and ROP incidence. Multiple linear regression analysis was used to determine the key influencing factors affecting ROP incidence. *P* < 0.05 was considered statistically significant.

## 3 Results

### 3.1 Demographic and clinical characteristics

The study cohort comprised 603 premature infants, including 317 males (52.6%) and 286 females (47.4%). The mean ± standard deviation (SD) gestational age at birth was 32 ± 2.3 weeks (range: 23–36 weeks), and the mean birth weight was 1,600 ± 470 g (range: 450–3,600 g). The demographic and clinical characteristics of the participants, stratified by the five consecutive 2-year study intervals, are summarized in Table 1.

**Table 1:** Demographic characteristics of participants.

Characteristic	2014–2015	2016–2017	2018–2019	2020–2021	2022–2023
Birth weight, mean ± SD (g)	1,649 ± 470	1,600 ± 320	1,570 ± 340	1,470 ± 440	1,510 ± 320
Gestational age, mean ± SD (weeks)	32 ± 2.3	31 ± 2.2	32 ± 2	30 ± 1.9	31 ± 2.1
Sex, *n* (%)					
Male	57 (56.4)	62 (50.8)	67 (53.6)	68 (50.7)	63 (52.1)
Female	44 (43.6)	60 (49.2)	58 (46.4)	66 (49.3)	58 (47.9)
Birth weight (g), *n* (%)					
≤1,250	16 (15.8)	23 (18.9)	27 (21.6)	31 (23.1)	26 (21.5)
1,251–1,500	63 (62.4)	72 (59.0)	69 (55.2)	79 (59.0)	71 (58.7)
≥1,500	22 (21.8)	27 (22.1)	29 (23.2)	24 (17.9)	24 (19.8)
Gestational age (weeks), *n* (%)					
≤28	6 (5.9)	9 (7.4)	10 (8.0)	9 (6.7)	13 (10.7)
28–32	76 (75.2)	89 (73.0)	98 (78.4)	107 (79.9)	93 (76.9)
≥32	19 (18.8)	24 (19.7)	17 (13.6)	18 (13.4)	15 (12.4)
Sepsis/bacteremia incidence, *n* (%)	29 (28.7)	30 (24.6)	23 (18.4)	18 (13.4)	10 (8.3)
Oxygen therapy compliance, *n* (%)	66 (65.3)	88 (72.1)	98 (78.5)	114 (85.2)	111 (91.7)
Breastfeeding rate, *n* (%)	49 (48.5)	67 (55.3)	79 (62.8)	94 (70.4)	95 (78.9)
Prenatal glucocorticoid use rate, *n* (%)	63 (62.4)	84 (68.8)	89 (71.7)	88 (65.6)	90 (74.4)
Total, *n*	101	122	125	134	121

### 3.2 Incidence and trends of ROP

A total of 116 premature infants were diagnosed with ROP, resulting in an overall incidence of 19.2% (116/603). Among these cases, 31 (5.1% of the entire cohort) were classified as Type 1 ROP. A strong inverse correlation was identified between birth weight/gestational age and ROP incidence: the lower the birth weight or gestational age, the higher the incidence of ROP, with a multiplicative increase observed. Specifically, the incidence of ROP was 1.6% in infants with a birth weight >1,500 g, 13.6% in those with 1,251–1,500 g, and 53.7% in those with ≤1,250 g. In terms of gestational age, the incidence was 4.3% for infants born at >32 weeks, 17.3% for those at 28–32 weeks, and 68.1% for those at ≤28 weeks.

Over the five consecutive 2-year intervals, the overall incidence rates of ROP were 22.8%, 21.3%, 19.2%, 17.2%, and 16.5%, respectively; the corresponding incidence rates of Type 1 ROP were 5.9%, 5.7%, 5.6%, 4.5%, and 4.2%. Both metrics exhibited a consistent downward trend throughout the study period.

For the subgroup of birth weight >1,500 g: the number of screenings in 2014–2015, 2016–2017, 2018–2019, 2020–2021, and 2022–2023 was 22, 27, 29, 24, and 24 respectively; the number of ROP cases was 1 (4.5%), 1 (3.7%), 1 (3.4%), 0 (0%), and 1 (4.2%) respectively; the number of Type 1 ROP cases was 1 (4.5%), 1 (3.7%), 0 (0%), 0 (0%), and 0 (0%) respectively. Cochran-Armitage test showed that the downward trend of Type 1 ROP incidence in this subgroup was statistically significant (*P* = 0.032).

For the subgroup of gestational age >32 weeks: the number of screenings in 2014–2015, 2016–2017, 2018–2019, 2020–2021, and 2022–2023 was 19, 24, 17, 18, and 15 respectively; the number of ROP cases was 1 (5.3%), 2 (8.3%), 0 (0%), 1 (4.2%), and 0 (0%) respectively; the number of Type 1 ROP cases was 0 (0%), 1 (4.2%), 0 (0%), 0 (0%), and 0 (0%) respectively. Cochran-Armitage test showed that the downward trend of Type 1 ROP incidence in this subgroup was statistically significant (*P* = 0.045).

Detailed data on ROP and Type 1 ROP incidence, stratified by time interval, birth weight, and gestational age, are summarized in Table 2.

**Table 2:** Incidence of ROP and type 1 ROP in Sichuan (2014–2023).

Variable	2014–2015	2016–2017	2018–2019	2020–2021	2022–2023
Overall incidence					
ROP (*n*, %)	23 (22.8)	26 (21.3)	24 (19.2)	23 (17.2)	20 (16.5)
Type 1 ROP (*n*, %)	6 (5.9)	7 (5.7)	7 (5.6)	6 (4.5)	5 (4.2)
ROP incidence stratified by birth weight					
≤1,250 g (screened *n*, ROP *n*, %)	16, 10 (62.5)	23, 14 (60.9)	27, 14 (51.9)	31, 16 (51.6)	26, 12 (46.2)
1,251–1,500 g (screened *n*, ROP *n*, %)	63, 12 (19.0)	72, 11 (15.2)	69, 9 (13.0)	79, 7 (8.9)	71, 7 (9.9)
>1,500 g (screened *n*, ROP *n*, %)	22, 1 (4.5)	27, 1 (3.7)	29, 1 (3.4)	24, 0 (0.0)	24, 1 (4.2)
>1,500 g (screened *n*, Type 1 ROP *n*, %)	22, 1 (4.5)	27, 1 (3.7)	29, 0 (0.0)	24, 0 (0.0)	24, 0 (0.0)
ROP incidence stratified by gestational age					
≤28 weeks (screened *n*, ROP *n*, %)	6, 5 (83.3)	9, 7 (77.7)	10, 7 (70.0)	9, 6 (66.7)	13, 7 (53.8)
28–32 weeks (screened *n*, ROP *n*, %)	76, 17 (22.4)	89, 17 (19.1)	98, 17 (17.3)	107, 16 (15.0)	93, 13 (14.0)
>32 weeks (screened *n*, ROP *n*, %)	19, 1 (5.3)	24, 2 (8.3)	17, 0 (0.0)	18, 1 (4.2)	15, 0 (0.0)
>32 weeks (screened *n*, Type 1 ROP *n*, %)	19, 0 (0.0)	24, 1 (4.2)	17, 0 (0.0)	18, 0 (0.0)	15, 0 (0.0)
Type 1 ROP incidence stratified by birth weight					
≤1,250 g (screened n, type 1 ROP *n*, %)	16, 3 (18.8)	23, 4 (17.4)	27, 5 (18.5)	31, 5 (16.1)	26, 4 (15.4)
1,251–1,500 g (screened *n*, Type 1 ROP *n*, %)	63, 2 (3.2)	72, 2 (2.8)	69, 2 (2.9)	79, 1 (1.3)	71, 1 (1.4)
Type 1 ROP incidence stratified by gestational age					
≤28 weeks (screened *n*, Type 1 ROP *n*, %)	6, 4 (66.7)	9, 5 (55.6)	10, 5 (50.0)	9, 4 (44.4)	13, 4 (30.8)
28–32 weeks (screened *n*, Type 1 ROP *n*, %)	76, 2 (2.6)	89, 1 (1.1)	98, 2 (2.0)	107, 2 (1.9)	93, 1 (1.1)

### 3.3 Results of decision analysis model

The results of the decision analysis model showed that under different birth weight screening thresholds, the relevant indicators are as follows:

It can be seen from Table 3 that when the birth weight threshold is adjusted to <1,500 g, the detection rate of Type 1 ROP is still as high as 96.8%, which can basically ensure that most Type 1 ROP cases are detected. At the same time, the absolute screening cost is reduced from 1,400 yuan/case (current threshold) to 945 yuan/case (a reduction of 32.5%), and the false positive rate is reduced by 28.3%. Notably, the follow-up cost (900 yuan/case) is three times the single fundus examination fee (300 yuan/case), which was based on the minimum follow-up requirements outlined in the Chinese Guidelines for Screening of Retinopathy of Prematurity (2014) and may oversimplify actual screening costs in real-world clinical practice where follow-up frequency is stratified by disease severity. This adjustment significantly improves the screening efficiency and cost-effectiveness. When the threshold is <1,750 g, the detection rate is slightly higher (98.7%), but the absolute cost (1,096.2 yuan/case) and cost reduction amplitude are less obvious than those of <1,500 g.

**Table 3:** Simulation results of different screening thresholds.

Screening threshold (birth weight)	Detection rate of type 1 ROP (*n*, %)	Screening cost (relative value, *n*)	Screening cost (absolute value, yuan/case)	False positive rate (*n*, %)
<2,000 g (current)	31 (100.0)	100	1,400	112 (18.6)
<1,750 g	30 (98.7)	78.3	1,096.2	86 (14.2)
<1,500 g	30 (96.8)	67.5	945	80 (13.3)

The screening cost is expressed as a relative value with the current threshold cost as 100.0. The absolute cost includes fundus examination fee (300 yuan/case, single time), personnel cost (200 yuan/case), and follow-up cost (900 yuan/case, cumulative for 3 re-examinations).

### 3.4 Analysis of influencing factors for incidence decline

Correlation analysis showed that sepsis/bacteremia incidence (r = 0.83, *P* < 0.01) was significantly positively correlated with ROP incidence, indicating that the higher the incidence of sepsis/bacteremia, the higher the ROP incidence. Oxygen therapy compliance (r = −0.89, *P* < 0.01) and breastfeeding rate (r = −0.76, *P* < 0.05) were significantly negatively correlated with ROP incidence, while prenatal glucocorticoid use rate had no statistically significant correlation with ROP incidence (r = −0.21, *P* = 0.35).

Multiple linear regression analysis was performed with ROP incidence as the dependent variable and the above four clinical practice indicators as independent variables. The results showed that sepsis/bacteremia incidence (*β* = 0.45, *P* < 0.01), oxygen therapy compliance (*β* = −0.42, *P* < 0.01), and breastfeeding rate (*β* = −0.28, *P* < 0.05) were all important factors affecting ROP incidence, and together explained 86.3% of the variation in ROP incidence (R² = 0.863, *P* < 0.01). Prenatal glucocorticoid use rate was not an independent influencing factor (*β* = −0.08, *P* = 0.35).

## 4 Discussion

The incidence of ROP varies significantly by region, primarily due to differences in premature infant survival rates, neonatal care standards, and treatment protocols across areas (5). In mainland China, the overall incidence of ROP is reported to range from 8.8% to 13.8%, while the incidence of Type 1 ROP requiring clinical intervention is between 3.4% and 4.9% (6–11). In our single-center retrospective study of 603 premature infants in Sichuan, the overall ROP incidence was 19.2% and the Type 1 ROP incidence was 5.1%, both slightly higher than the national average. This discrepancy is attributed to the tertiary referral nature of our institution, which receives a high volume of critically ill premature infants transferred from surrounding regions, resulting in a cohort with a higher proportion of high-risk cases, and this characteristic also limits the direct generalizability of our study results to the general preterm infant population in China. Consistent with prior literature (6), our findings reconfirm that low birth weight and low gestational age are the primary risk factors for ROP, with a strong inverse correlation between these variables and disease incidence. The risk of ROP increases multiplicatively as birth weight and gestational age decrease: infants with a birth weight ≤1,250 g had an ROP incidence of 53.7%, compared to 13.6% for those with 1,251–1,500 g and 1.6% for those >1,500 g; similarly, infants born at ≤28 weeks of gestation had a 68.1% incidence, versus 17.3% for 28–32 weeks and 4.3% for >32 weeks. This persistent association underscores the rationale for using birth weight and gestational age as the core criteria for ROP screening globally.

Notably, our stedy reveal a significant downward trend in ROP incidence in Sichuan over the past decade, consistent with reports from other regions with improving neonatal care (6, 14–16). From 2014–2015 to 2022–2023, the overall ROP incidence decreased from 22.8% to 16.5%, and Type 1 ROP incidence declined from 5.9% to 4.2%. A similar trend was observed in Hubei Province, where ROP incidence dropped from 16.61% in 2012 to 8.8% in 2022, driven by enhanced clinician awareness, standardized oxygen therapy, and improved screening compliance (6, 14). This global decline in ROP incidence, fueled by advancements in neonatal intensive care, highlights the need to periodically re-evaluate and refine screening thresholds to optimize cost-effectiveness (13).

A critical finding of our study is the absence of Type 1 ROP cases in infants with a birth weight >1,500 g or gestational age >32 weeks after 2018. Cochran-Armitage test confirmed that the downward trend of Type 1 ROP incidence in these two subgroups was statistically significant (*P* < 0.05), indicating a shifting risk profile for severe ROP toward smaller and less mature infants. This shift is likely a result of improved prenatal and neonatal care, which has reduced the risk of severe ROP in relatively mature and heavier premature infants. These improvements in clinical practice (e.g., infection control, standardized oxygen therapy) have not only reduced overall ROP incidence but also reshaped the risk profile of severe ROP, laying the foundation for optimizing screening thresholds. Notably, ROP screening guidelines are dynamic and revised every 5–10 years based on local epidemiology (5, 12), yet China's current criteria have remained unchanged for two decades (3)—a mismatch addressed by our findings. For context, screening criteria in Hong Kong, China and the United Kingdom stipulate a birth weight <1,501 g or a gestational age ≤31 + 6 weeks, while the United States employs a gestational age ≤30 weeks or a birth weight ≤1,500 g (12, 13). The United Kingdom recently updated its guidelines in 2022, further emphasizing the need for timely revisions aligned with clinical advancements (13, 17).

The results of our decision analysis model further support the tentative feasibility of adjusting ROP screening thresholds for tertiary medical centers in Sichuan. When the birth weight screening threshold was adjusted to <1,500 g, the Type 1 ROP detection rate remained as high as 96.8%, ensuring that most severe cases were not missed. Meanwhile, this adjustment reduced screening costs by 32.5% (from 1,400 yuan/case to 945 yuan/case) and lowered the false positive rate by 28.3% (from 18.6% to 13.3%). In contrast, adjusting the threshold to <1,750 g yielded a slightly higher detection rate (98.7%) but a less substantial reduction in costs (21.7%) and false positive rates (23.7%), making it less cost-effective. Due to the small number of Type 1 ROP cases in subgroups with birth weight >1,500 g or gestational age >32 weeks, the conclusion about the screening threshold adjustment should be interpreted with extreme caution. Considering the uneven distribution of medical resources in Sichuan, we tentatively propose a stratified screening strategy: economically developed areas with sufficient resources (e.g., Chengdu Plain) can adopt the <1,500 g threshold to improve screening efficiency, while relatively underdeveloped areas (e.g., western Sichuan Plateau) may temporarily retain the current <2,000g threshold to avoid missing high-risk cases, with gradual adjustments as medical conditions improve. Any adjustment of national ROP screening guidelines requires validation through large-scale, prospective, multicenter population-based studies across different provinces in China.

Correlation and regression analyses identified key factors influencing the decline in ROP incidence. Sepsis/bacteremia was an independent risk factor for ROP (*β* = 0.45, *P* < 0.01), with a strong positive correlation between sepsis/bacteremia incidence and ROP incidence (r = 0.83, *P* < 0.01). Severe infections like sepsis trigger systemic inflammatory responses in premature infants, leading to retinal vascular endothelial damage and abnormal proliferation, thereby increasing ROP risk (18, 19). The incidence of sepsis/bacteremia in our cohort decreased from 28.7% in 2014–2015 to 8.3% in 2022–2023, likely contributing to the decline in ROP incidence. In contrast, oxygen therapy compliance (r = −0.89, *P* < 0.01) and breastfeeding rate (r = −0.76, *P* < 0.05) were significantly negatively correlated with ROP incidence and emerged as protective factors (*β* = −0.42, *P* < 0.01 and *β* = −0.28, *P* < 0.05, respectively). The substantial improvement in oxygen therapy compliance (from 65.3% to 91.7%) reduced retinal vascular damage caused by inappropriate oxygen use, while breastfeeding provided essential nutrients and bioactive substances that enhance immunity and promote retinal vascular maturation (1, 6). Interestingly, prenatal glucocorticoid use rate showed no significant correlation with ROP incidence (r = −0.21, *P* = 0.35), possibly due to its high utilization rate (over 68% after 2018) leading to a weakened marginal protective effect, or the small sample size of extremely premature infants (47 cases, 7.8% of the total) affecting statistical power.

These findings provide targeted implications for clinical practice to further reduce ROP incidence: strengthening neonatal infection prevention and control through standardized antibiotic use, enhanced hand hygiene, and optimized isolation measures; improving oxygen therapy standardization via medical staff training; and promoting breastfeeding with supportive measures for mothers. Importantly, clinical management should focus on comprehensive care rather than over-reliance on prenatal glucocorticoids for ROP prevention.

This study has several limitations. First, the single-center, retrospective design may limit generalizability, as our cohort includes a high proportion of critically ill transferred infants, and the proposed <1,500 g screening threshold is a Sichuan-specific regional recommendation for tertiary medical centers, which cannot be directly extended to other regions or the general preterm infant population in China. Multicenter, population-based studies across different provinces in China are urgently needed to validate our findings and formulate national-level ROP screening guidelines. Second, while the sample size is adequate for temporal trend analysis, larger multi-center prospective studies are needed to validate these findings across diverse healthcare settings in China, especially to verify the clinical applicability of the adjusted screening threshold due to the small number of Type 1 ROP cases in some subgroups. Third, the decision analysis model's parameters (e.g., screening cost components) are based on local medical resource conditions and may require adjustments for other regions; in addition, the model assumed an average of three follow-up visits for all infants based on the national guideline requirements, which oversimplifies actual screening costs as follow-up frequency in clinical practice is stratified according to the severity of ROP lesions, and future studies should incorporate more sophisticated patient-level cost models to validate our preliminary findings. Despite these limitations, our study provides robust evidence of a declining ROP incidence in Sichuan Province, highlights a potential mismatch between current screening criteria and contemporary epidemiological patterns, and clarifies key drivers of the incidence decline, offering valuable guidance for clinical practice and future revisions to national screening guidelines.

In conclusion, the incidence of ROP in Sichuan Province has decreased significantly over the past decade. Type 1 ROP has not been observed in infants with a birth weight >1,500 g or gestational age >32 weeks since 2018, with a statistically significant downward trend in these subgroups. Adjusting the birth weight screening threshold to <1,500 g may enhance screening cost-effectiveness for tertiary medical centers in Sichuan while maintaining a high detection rate for severe ROP, but this conclusion should be interpreted with caution due to the single-center study design and small sample size of Type 1 ROP cases in some subgroups. Sepsis and other infectious diseases are key risk factors, while standardized oxygen therapy and breastfeeding are important protective factors for ROP in Sichuan. Larger multicenter prospective population-based studies across different regions of China are urgently needed to validate the proposed screening threshold and provide high-quality evidence for the revision of regional or national ROP screening guidelines.

## Data Availability

The raw data supporting the conclusions of this article will be made available by the authors, without undue reservation.

## References

[1] Fundus Disease Group of the Ophthalmology Branch of the Chinese Medical Association. Screening guidelines for retinopathy of prematurity in China (2014). Chin J Ophthalmol. (2014) 50(12):933–935. 10.3760/cma.j.issn.0412-4081.2014.12.017

[2] The Ophthalmology Group of the Pediatric Branch of the Chinese Medical Association. Expert consensus on the treatment specifications of retinopathy of prematurity. Chin J Ocul Fundus Dis. (2022) 38(1):4. 10.3760/cma.j.cn511434-20211119-00647

[3] Li XX. Characteristics and screening guidelines of retinopathy of prematurity in China. Chin J Ocul Fundus Dis. (2004) 20(6):53–55. 10.3760/j:issn:0412-4081.2005.08.019

[4] Fundus Disease Group of the Ophthalmology Branch of the Chinese Medical Association, Fundus Disease Special Committee of the Ophthalmologists Branch of the Chinese Medical Association. Expert consensus on the classification and treatment of retinopathy of prematurity in China (2023). Chi J Ocul Fundus Dis. (2022) 38(9):720–727. 10.3760/cma.j.cn511434-20230815-00346

[5] Zhu BD Li SJ. Current status of screening and treatment for retinopathy of prematurity in China. Chin J Strabismus Pediatr Ophthalmol. (2019) 27(3):44–47. 10.3969/J.ISSN.1005-328X.2019.03.016

[6] Yuan H Guo Z Liu SF Yin FR Xiang SH Jie H Analysis of RetCam III fundus examination results in 1111 premature infants and infants in Hubei province. Chin J Strabismus Pediatr Ophthalmol. (2022) 30(04):33–36. 10.3969/J.ISSN.1005-328X.2022.04.010

[7] Chen JG Xiong LC Ge P Lin XM Lin SH Liu GH. Screening results and risk factor analysis of retinopathy of prematurity in 617 premature infants in Fuzhou area. J Clin Ophthalmol. (2019) 27(06):546–548. CNKI:SUN:LCYZ.0.2019-06-028

[8] Shan HD Bu J Kamili N Zhang F Huang X Ni YQ Screening and analysis of retinopathy of prematurity in Kashgar, Xinjiang. Chin J Ophthalmol Otorhinolaryngol. (2018) 18(6):3. 10.14166/j.issn.1671-2420.2018.06.007

[9] Wang YS Zhang ZF Li MH Zhang P Liu XY. Preliminary results of screening for retinopathy of prematurity in Xi'an area. Chin J Ophthalmol. (2010) 46(2):119–123. 10.3760/cma.j.issn.0412-4081.2010.02.006 20388344

[10] Jin SX Xu JT Xiao YS Xia HY Wu SQ Zhao SP Analysis of the incidence rate of retinopathy of prematurity in 1010 premature infants or low birth weight infants in Kunming children’s hospital and its influencing factors. Chin J Ocul Fundus Dis. (2016) 32(3):314–316. 10.3760/cma.j.issn.1005-1015.2016.03.022

[11] Ma XR Zhang Q Kang H Li XP Zhou ZJ Wang J. Screening results and risk factor analysis of retinopathy of prematurity in 310 premature infants in Qinghai province. Chin J Ocul Fundus Dis. (2017) 33(6):631–632. 10.3760/cma.j.issn.1005-1015.2017.06.018

[12] Mi XS Zhao PQ. Overview of ROP screening standards in Various countries around the world. Chin J Pract Ophthalmol. (2006) 24(9):879–882. 10.3760/cma.j.issn.1006-4443.2006.09.001

[13] Dong XY Li JZ Luo KR Tang J Mu DZ. Interpretation of the 2022 updated version of the guidelines for the screening and treatment of retinopathy of prematurity in the United Kingdom. Chin J Contemp Pediatr. (2024) 26(05):437–443. 10.7499/j.issn.1008-8830.2311073 PMC11135053 38802901

[14] Su Y Chen CZ Li L Yu XX Zheng HM Zhou LH Screening results and related factors analysis of retinopathy of prematurity in Hubei region. Chin J Ocul Fundus Dis. (2012) 28:4. 10.3760/cma.j.issn.1005-1015.2012.01.014

[15] Chow PP Yip WW Ho M Lok JY Lau HH Young AL. Trends in the incidence of retinopathy of prematurity over a 10-year period. Int Ophthalmol. (2019) 39(4):903–909. 10.1007/s10792-018-0896-0 29907928

[16] Dhaliwal C Fleck B Wright E Graham C McIntosh N. Incidence of retinopathy of prematurity in Lothian, Scotland, from 1990 to 2004. Arch Dis Child Fetal Neonatal Ed. (2008) 93(6):F422–F426. 10.1136/adc.2007.134791 18463118

[17] Wilkinson AR Haines L Head K Fielder AR. Guideline development group of the royal college of paediatrics and child health; royal college of ophthalmologists; British association of perinatal medicine. UK retinopathy of prematurity guideline. Eye. (2009) 23(11):2137–2139. 10.1038/eye.2008.128 18836408

[18] El Emrani S Van der Meeren LE Jansen EJS De Jong E Verboven S Carmeliet P Early-onset sepsis as an early predictor for retinopathy of prematurity: a meta-analysis. Am J Perinatol. (2025) 42(3):387–94. 10.1055/a-2369-6690 39029916 PMC11793952

[19] Glaser K Hartel C Klingenberg C Wenzel D Borschke K Wiese L Neonatal sepsis episodes and retinopathy of prematurity in very preterm infants. JAMA Netw Open. (2024) 7(7):e2423933. 10.1001/jamanetworkopen.2024.23933 39052290 PMC11273231

